# Examination of youth sexual and reproductive health transitions in Nigeria and Kenya using longitudinal data

**DOI:** 10.1186/s12889-017-4039-8

**Published:** 2017-01-31

**Authors:** Ilene S. Speizer, David Guilkey, Lisa M. Calhoun, Meghan Corroon, Rick O’Hara

**Affiliations:** 1 Department of Maternal and Child Health, Gillings School of Global Public Health, University of North Carolina at Chapel Hill, Chapel Hill, NC USA; 2 Department of Economics, University of North Carolina at Chapel Hill, Chapel Hill, NC USA; 3 Carolina Population Center, University of North Carolina at Chapel Hill, Chapel Hill, NC USA

**Keywords:** Youth, Nigeria, Kenya, Urban, Sexual initiation, Marriage, Pregnancy

## Abstract

**Background:**

The adolescent (ages 15–19) and young adult (ages 20–24) years are a crucial time as many sexual and reproductive health (SRH) transitions take place in these years. The study of youth SRH transitions in sub-Saharan Africa is limited due to a paucity of longitudinal data needed to examine the timing and circumstances of these transitions.

**Methods:**

This paper uses recently collected longitudinal data from select urban areas in Kenya and Nigeria that include a large youth sample at baseline (2010/2011) and endline (2014). We control for unobserved heterogeneity in our modelling approach to correct for selectivity issues that are often ignored in similar types of analyses.

**Results:**

We demonstrate that the transition patterns (i.e., sexual initiation, first marriage, and first pregnancy/birth) differ within and across the urban areas and countries studied. Urban Kenyan youth have more premarital sex and pregnancy than youth from the Nigerian cities. Further analyses demonstrate that more educated and wealthier youth transition later than their less educated and poorer counterparts.

**Conclusions:**

The findings from this study can be used to inform programs seeking to serve young people based on their varying reproductive health needs in different contexts over the adolescent and young adult years.

## Background

In 2015, nearly one-fifth of the population in sub-Saharan Africa (SSA) was young people between the ages of 15–24 [1]. This is a large population that will undertake important sexual and reproductive health transitions over the next decade. Many key sexual and reproductive health (SRH) life transitions including sexual initiation, first marriage, and first birth occur in the adolescent (ages 15–19) or young adult (20–24) years. Among SSA young people who are in school, these transitions may be delayed relative to young people who are no longer in school [2, 3].

Adolescents account for 11% of all births worldwide and 16% of births in SSA; women 20–24 account for another quarter of births in SSA [4]. Most adolescent and young adult births are to women in union (married or living with a partner) and thus these births are generally reported as intended [5]. Prevention of early transitions including early or premarital sex, early marriage, and early and unintended pregnancy is important for attaining the Sustainable Development Goals as well as ensuring that every young person leads a healthy life [6, 7]. In particular, early transition to sex, marriage, and pregnancy can lead to negative health and social outcomes [8, 9]. For example, young women having early and unprotected sex are at greater risk of HIV and complications of an early or unintended pregnancy. Further, the social consequences of early transitions include school dropout, lower educational attainment and greater risk of poverty which may have negative long-term effects on births to the young mothers [6, 7].

Examining adolescent and young adult transitions using a life-course perspective permits a better understanding of sexual initiation, within or outside of marriage; the transition to first marriage; and the transition to first pregnancy/birth and their connections to earlier and later phases of one’s life [10]. Decisions that are made during the adolescent or young adult years influence the long-term health and well-being of a young person, her family members and her friends [11]. Numerous individual-level factors, including education level, religion, employment status, school enrollment, age, and family living arrangement influence the decisions that young people make [12]. In addition, there are additional levels of influence on a young person’s reproductive health transitions including structural factors at the community or institutional levels, and cultural and family-level factors that affect decision-making [7, 13]. Notably, these decisions are also being made within a policy and regulatory environment that may be more or less supportive of behaviors such as delayed marriage or contraceptive use at an early age or out of union [7, 13].

Few research studies on youth in developing countries use longitudinal data to examine simultaneously the sexual and reproductive health transitions of young people. Recent studies using longitudinal data of youth have demonstrated the above listed multiple-level influences on sexual initiation [14, 15], and on pregnancy experience as it relates to school enrollment/continuation or school drop-out [16]. Earlier studies using longitudinal or retrospective data have examined the association between school enrollment and performance and delayed sexual initiation as well as avoidance of an unintended pregnancy [16, 17]. Longitudinal data have also been used for evaluations of the impact of social welfare programs for young people as they relate to staying in school, delaying first marriage, reducing the risk of HIV, and delayed childbearing [18–20]. Use of longitudinal data to examine key sexual and reproductive health transitions provides a rich approach to understanding sexual and reproductive health risk-taking among young people and can be used to support the design of comprehensive programs to prevent unintended pregnancy risk and long-term health and well-being of the next generation.

Our paper contributes to this prior research by simultaneously examining the timing of sexual initiation, first marriage, and first pregnancy using recently collected longitudinal data from select urban areas in Kenya and Nigeria. The advantage of longitudinal data in this case is that we are able to more accurately examine the timing of key sexual and reproductive health transitions (first sex, first marriage, and first pregnancy/birth) that take place in the follow-up period; this leads to less reliance on retrospective recall data that is often misreported. Our survey includes a large, representative sample of female youth at baseline in 2010/2011 and those who are followed over four years to the endline in 2014. The objectives of this paper are to: a) discuss differences across study contexts in adolescent and young adult transitions to first sex, marriage, and pregnancy/birth; and b) examine demographic factors associated with transitions to first sex, first marriage, and first pregnancy/birth in the varying study contexts.

### Study context

#### Nigeria

This study focuses on six cities in Nigeria. The cities were selected purposely to include large sites (with a population of about a million, with one exception – the capital city of Abuja). Nigeria is a large and diverse country with numerous ethnic groups and religions spread across the regions of the country. The study cities come from the North Central region (Ilorin and Abuja), the North West region (Kaduna and Zaria), the South South region (Benin City), and the South West region (Ibadan). Notably, the north of the country is generally more conservative with social norms that are less supportive of female education, contraceptive use, and delayed marriage, whereas the southern regions tend to be somewhat more liberal with more female education, delayed marriage, and contraceptive use. This means that the study cities included provide a varied, but not necessarily representative, picture of urban Nigeria. See the baseline study report for descriptive characteristics of the six Nigerian study cities included for this study [21].

#### Kenya

The five study cities in Kenya include the three largest cities (in order of size: Nairobi, Mombasa, and Kisumu) and two smaller cities Machakos (near Nairobi) and Kakamega (near Kisumu). Across the five study cities, Mombasa is the most dissimilar to the others with the largest population that is Muslim (about 1/3 of our sample at baseline was Muslim in this city). There are 42 ethnic groups in Kenya, with regional differences in the distribution of each ethnic group. Ethnic groups often have distinct cultural beliefs and practices that may be associated with the sexual and reproductive health behaviors of interest. Among the study cities, Kisumu, Kakamega, and Machakos each are dominated by a different ethnic group and therefore city-level differences will somewhat reflect these ethnic group effects. The two other major cities (Nairobi and Mombasa) are more diverse in terms of ethnic group make up. See the baseline report for descriptive characteristics of the five Kenya cities included in this study [22].

## Methods

The data for this paper come from the Measurement, Learning & Evaluation (MLE) project. The MLE project is funded by the Bill & Melinda Gates Foundation (BMGF) and was tasked with evaluating the Urban Reproductive Health Initiative (URHI) undertaken in select cities in India, Kenya, Nigeria, and Senegal. The focus of this paper is on Nigeria and Kenya where an earlier study demonstrated that premarital sexual activity is common, although more common in Kenya than Nigeria [23]. The MLE project designed a longitudinal evaluation that followed baseline respondents (2010/2011) four years later at endline (2014). At baseline, a representative sample of women from each of the cities was selected.

In each city, a two-stage sampling approach was used. In the first stage, a recent census was used to define primary sampling units (PSUs). A representative sample of PSUs was then selected with the number of PSUs determined based on expected response rates from previous surveys of urban women in each country. In the second stage, within each selected PSU, a random sample of households (41 in Nigeria and 30 in Kenya) was selected. In selected households all women between the ages of 15–49 who spent the previous night in the household were approached and asked for verbal consent to be interviewed. All study procedures were approved by the Institutional Review Board (IRB) at the University of North Carolina at Chapel Hill and in-country IRBs in Nigeria and Kenya (the Nigeria Health and Research Ethics Committee and the Kenya Medical Research Institute Ethical Review Committee, respectively).

A total of 16,144 and 8,932 women were interviewed at baseline in Nigeria and Kenya, respectively. At baseline, 35 and 40% of the surveyed sample in Nigeria and Kenya were youth (age 15–24). At endline, all women who were usual residents in the household at baseline were eligible for follow-up interview. Field teams tracked eligible respondents who were still living within any of the study cities; women who moved outside the study cities were lost to follow-up. Within two weeks of being tracked, interviewing teams returned to the women’s households to undertake the individual-level endline interview. In total, 62.8% of eligible baseline youth (ages 15–24) from Nigeria were tracked and interviewed. In Kenya, 48.8% of eligible youth were tracked and interviewed. In Kenya there was high mobility of all women. Further, in Kenya, youth lost to follow-up were more likely to be sexually experienced, married and ever pregnant at baseline; there was no difference in these behaviors at baseline between the full Nigeria sample and the endline sample of youth surveyed at both time points (matched sample). We discuss the implications for the study findings of the high loss to follow-up in the limitations section. In this paper, we use the full information on baseline SRH indicators among all youth interviewed at baseline in the age group 15–24; in addition, for those interviewed four years later, we also include their endline indicators.

The dependent variables in this analysis are the age at first sex, the age at first marriage, and the age at first pregnancy. The variables were constructed as follows: At baseline and endline all women were asked their age at first sex (with an option “never had sex”). For age at first marriage, among respondents who had ever been married, a question was asked on age at first marriage. Finally, using information from the birth history or whether the woman is currently pregnant for women who never had a prior live birth, we create the age at first pregnancy. We backdate the data so that each woman enters the analysis at age 10 to eliminate left censoring in the data since almost all women experienced the transitions after age 10. Women contribute years to each of the events, starting at age 10, and ending at their baseline age (if lost to follow-up at endline) or at their endline age, if interviewed at endline. Women are coded as zero for each of the dependent variables for each age (year) prior to her transition to sex, marriage or pregnancy. At the age where the SRH transition takes place, the women are coded one. If a woman has not undergone the SRH transition by her baseline age (if lost to follow-up) or her endline age (if interviewed at endline) she is right censored in the model. Each woman has a separate observation for each age (year) in the model and women can contribute a maximum of 19 years (ages 10–28).

**Fig. 1:**
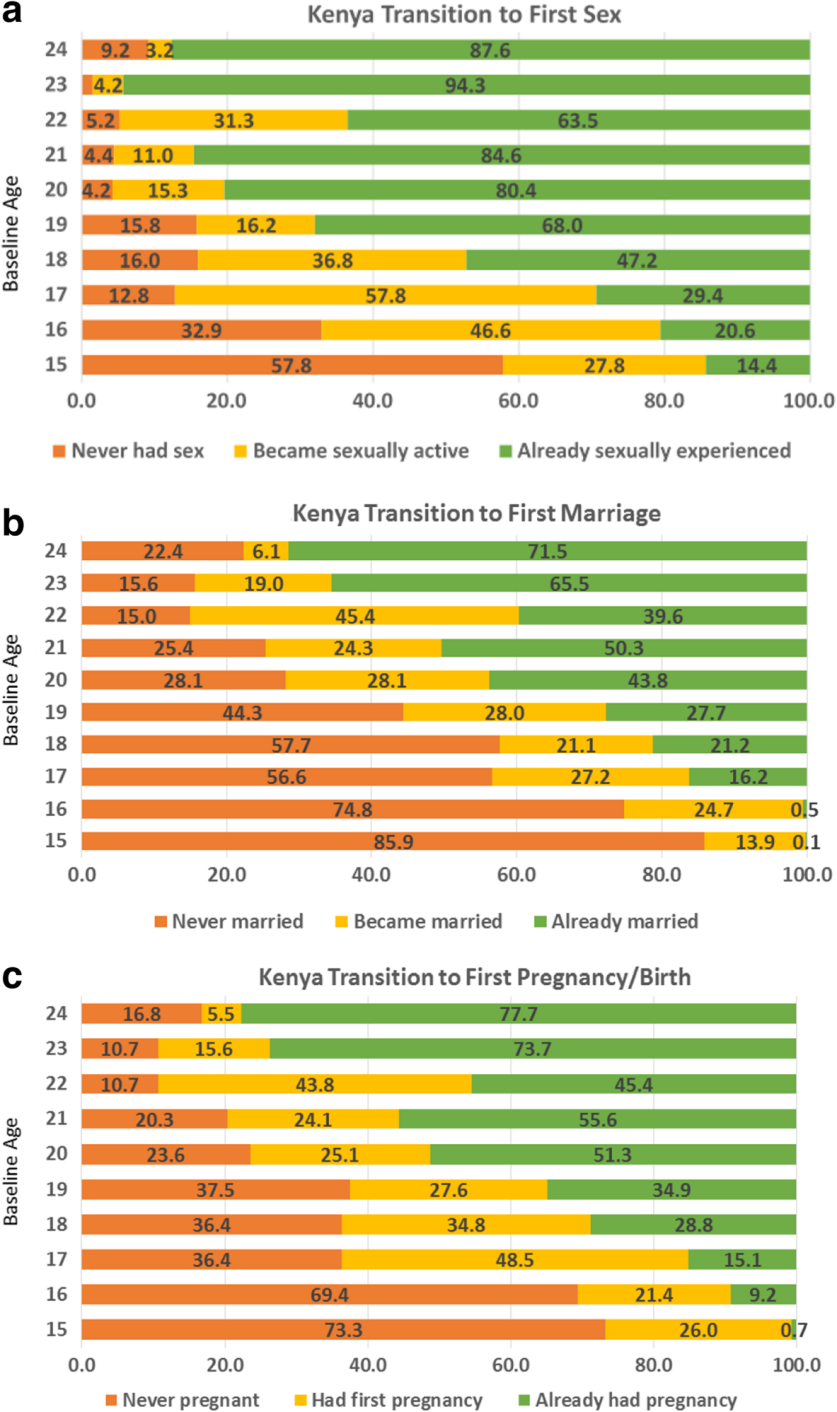
Kenya transitions between baseline and endline; **a** transition to first sex; **b** transtion to first marriage; **c** transition to first pregnancy/birth

**Fig. 2:**
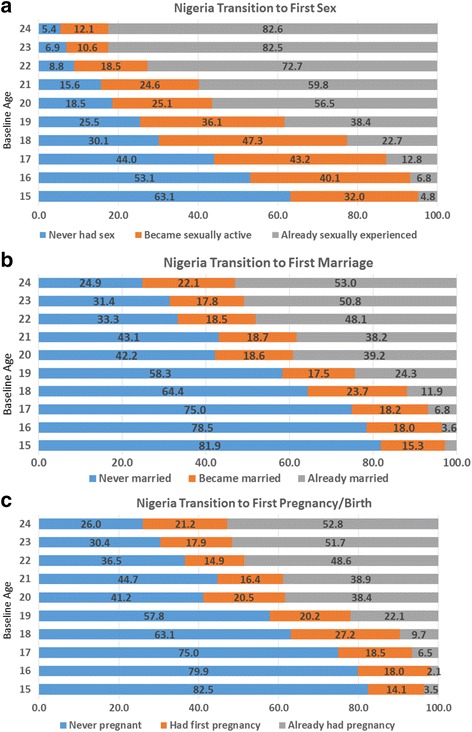
Nigeria transitions between baseline and endline

For descriptive purposes, we also examine the transitions to first sex, first marriage, and first pregnancy/birth among women ages 15–24 who were interviewed at both time points (matched sample). All women were asked at baseline and endline their age at first sex; these variables were used to create a four category variable of: 1) never had sex by endline; 2) had first sex between baseline and endline; 3) already had first sex at baseline; and 4) inconsistent. Likewise, a question about marital status at baseline and endline was used to create the marriage transition variable: 1) never married by endline; 2) became married between baseline and endline; 3) already married at baseline; and 4) inconsistent. Finally, the questions on pregnancy experience and timing of first pregnancy were used to create the pregnancy transition variable: 1) never pregnant by endline; 2) became pregnant between baseline and endline; 3) already had first pregnancy at baseline; and 4) inconsistent. The distributions of these variables can be seen in Fig. 1a–c for Kenya and Fig. 2a–c for Nigeria.^1^

To examine the demographic factors associated with SRH transitions, we include a number of exogenous factors that have been found to be related to sexual initiation, early marriage, and early pregnancy [12]. These include: age (starting from age 10) and age squared which are time varying variables in the model, education level, city, religion, whether or not the woman is currently a student at baseline or endline (Kenya only), religiosity (strongly religious versus somewhat religious/not religious), whether she reads newspapers, reads magazines, listens to the radio, watches television, whether she has visited another city in the last 12 months (urban mobility), whether she visited a rural area in the last 12 months (rural mobility), wealth, slum status (Kenya only), and an interaction between age and whether or not she is currently a student (Kenya only). In these models, the city variable captures some of the cultural distinctions across the cities. This is particularly relevant for Nigeria where there are large disparities in beliefs and behaviors in the different regions and in Kenya for the smaller cities. Note that most of these variables are coded based on the baseline values with the exception of education. At baseline and endline, respondents were asked the number of completed years of education and whether or not they are currently attending school (Kenya only). From these responses, we construct two time varying education variables: whether or not the respondent is currently in school and completed years of education at each age. The construction of these variables is based on the assumption that each respondent started primary school at age 6 and stayed in school continuously until she reached her terminal level of education. This, of course, can be right censored by the date of the endline interview if the respondent is still in school at endline.

We model the three transitions, the timing to first sex, marriage, and pregnancy, as discrete time hazard models of the following form:

1 $$ \ln \left[\frac{P\left({E}_{ij t}=1\Big|{E}_{ij, t-1}=0\right)}{P\left({E}_{ij t}=0\Big|{E}_{ij, t-1}=0\right)}\right]={X}_{ij t}\beta +{Z}_{ij}\alpha +{C}_j\gamma +{\lambda}_j+{\mu}_{ij}. $$

Where the dependent variable is the log odds that respondent *i* from community *j* experienced the transition at time *t*, given that she had not experienced the transition prior to time *t*. The *X*’s represent time varying individual level variables such as level of education, the *Z*’s represent time invariant individual variables such as the respondent’s religion, the *C*’s represent time invariant community level variables such as the respondent’s city of residence, and *β*, *α*, and *γ* represent unknown regression parameters to be estimated. In addition to an implicit uncorrelated error that varies both across individuals and through time, we allow for time invariant errors at both the individual and community levels, *μ*_*ij*_ and *λ*_*j*_. It is well known that when one estimates discrete time or continuous time hazards models without controls for this type of unobserved heterogeneity, biased and inconsistent parameter estimates could be obtained due to the fact that respondents drop from each model when they experience the event and the sample becomes increasingly self-selected [24]. Note that we model each transition as a function of variables that are assumed to be exogenous and so estimating each transition separately will yield consistent estimates of the model’s parameters as long as the two sources of time invariant heterogeneity are controlled. However, we estimate the three transitions jointly by allowing the individual and community level errors to be correlated to improve the precision of the parameter estimates.

To gain additional insights, we also ran two additional models to examine the timing of first marriage and first pregnancy after a woman has initiated first sex. In this case, the women self-select into the model for the marriage/pregnancy transitions upon engaging in first sex and they are coded zero until the age at first marriage/pregnancy. At the age of first marriage/pregnancy, respondents are coded to one (those women who have first sex and first marriage/pregnancy in the same year only contribute that one year to the model). Of course, some women are right-censored by the endline interview. The model includes the same exogenous factors but also includes a variable that measures the number of years since first sex; this variable is coded zero in the age when first sex occurred and each later age is augmented by one to count the number of years since first sex.

Because the timing of first sex is endogenous, we must jointly estimate the hazard equation for the timing of first sex with the timing of the marriage/pregnancy hazard conditional upon first sex having occurred. Thus, we estimate the first sex hazard as specified in Eq. (1) along with separate equations for the transition to marriage or pregnancy of the following form:

2 $$ \ln \left[\frac{P\left( P{M}_{ij t}=1\Big| P{M}_{ij, t-1}=0\right)}{P\left( P{M}_{ij t}=0\Big| P{M}_{ij, t-1}=0\right)}\right]=\delta \operatorname{s}\_ s e{x}_{ij t}+{X}_{ij t}{\beta}^{P M}+{Z}_{ij}{\alpha}^{P M}+{C}_j{\gamma}^{P M}+{\lambda}_j^{P M}+{\mu}_{ij}^{P M}. $$

Where the dependent variable is the log odds that respondent *i* from community *j* at time *t* experiences the event (marriage or pregnancy) conditional on the event not having occurred prior to time *t*. In this model, *s_sex* refers to the duration of time since the respondent had first sex and the other variables are as defined in Eq. (1). We do two sets of estimations: the transition to first sex and the transition to first marriage conditional on first sex having occurred and the transition to first sex and the transition to first pregnancy conditional on first sex having occurred. In each set of estimations we allow the time invariant individual and community heterogeneity to be correlated with the corresponding error in the other equation (see methodological appendix for more details of modeling approach).

## Results

Table 1 presents descriptive characteristics and control variables from the multivariate models for the urban sites across the two countries. The sample includes all women ages 15–24 at baseline; in Kenya, a smaller percentage of the sample is in the younger (15–19) age group than in the 20–24 age group; this may relate to the common practice of high school students attending boarding school and thus not being home during the interviews. More than half of the Kenya sample (59%) has secondary or higher education. One third has primary education. Two-thirds of the Kenya youth sample are Protestant and one quarter are Catholic and the remainder (14%) are Muslim or another religion. The overwhelming majority of the weighted sample is from Nairobi, however, the unweighted number of youth surveyed is more balanced across the other four cities. About one-fifth of the Kenya sample were in school at baseline. There is high exposure to television and radio in the Kenyan cities. At baseline, many Kenyan youth (55%) have visited a rural area in the last year and almost a third visited another urban area in the last year. Wealth is evenly split across five categories in the sample and about one-fifth of the sample is from a slum area in the weighted sample.

**Table 1:** Descriptive characteristics of female youth included in analysis of timing to first sex, first marriage, and first pregnancy/birth in urban sites of Kenya and Nigeria

	Kenya		Nigeria	
	%	Number (unweighted)	%	Number (unweighted)
		(*n* = 3,619)		(*n* = 5740)
Baseline age				
15	4.5	183	10.8	621
16	3.4	186	9.7	591
17	4.8	231	9.2	545
18	8.5	336	11.5	687
19	7.9	303	8.7	520
20	13.7	473	14.0	814
21	12.1	439	7.0	390
22	16.3	488	10.7	602
23	12.9	483	9.5	507
24	15.8	497	8.9	463
Education level				
None/Nursery/Quranic	2.8	81	5.5	366
Primary	36.1	1564	8.6	504
Post-primary/Junior secondary	2.4	87	15.0	932
Secondary/Senior secondary	44.4	1388	52.5	2937
College/University/Higher	14.3	490	18.4	940
Religion				
Catholic	23.2	863	4.9	230
Protestant	62.7	2418	42.4	2279
Muslim	14.1	335	52.6	3226
City				
Nairobi (Abuja - Nigeria)	71.1	1058	11.1	660
Mombasa (Benin City - Nigeria)	19.8	604	12.8	885
Kisumu (Ibadan - Nigeria)	6.0	736	16.0	856
Machakos (Ilorin - Nigeria)	1.4	699	16.6	822
Kakamega (Kaduna - Nigeria)	1.7	522	28.8	1137
na (Zaria - Nigeria)	na	na	14.8	1380
Student	18.5	758	na	na
Religiosity (% strongly religious)	52.8	1879	72.3	4249
Read newspapers (% yes)	41.6	1449	15.8	924
Read magazines (% yes)	32.4	1049	11.6	688
Listen to radio (% yes)	83.6	3126	70.3	4112
Watches TV (% yes)	77.3	2419	83.2	4692
Visits other cities in 12 months (% yes)	29.9	1180	24.5	1461
Visits rural areas in 12 months (% yes)	54.7	1849	14.0	888
Wealth group				
Poorest	71.1	1058	21.2	1133
Poor	19.8	604	18.3	1115
Middle	6.0	736	19.6	1098
Rich	1.4	699	20.2	1169
Richest	1.7	522	20.7	1225
Type of residence				
Slum area	20.4	1226	na	na
Formal/non-slum area	78.5	1936	na	na
Peri urban area	1.1	457	na	na

Note, all percentages are weighted, all n’s are unweighted

*na* is not available

The distributions of demographic characteristics for Nigeria are also presented in Table 1 for the full youth sample at baseline. The sample is evenly split across the age groups with some minor age heaping at age 20. More than 70% of youth in Nigeria has secondary or higher education. About half of the sample is Muslim and the other half is predominately Protestant with about 5% Catholic. Across the cities, the largest percentage is from Kaduna. Three quarters of the sample reports being strongly religious and a similarly high percentage reports listening to the radio or watching the television. Finally, there are greater numbers visiting other cities (25%) than visiting rural areas (14%).

### Transition to first sex, first marriage and first pregnancy/birth in Kenya and Nigeria

Figures 1a–c and 2a–c demonstrate the transitions to first sex, first marriage, and first pregnancy/birth in Kenya and Nigeria in the four year follow-up period for young women ages 15–24 at baseline interviewed at both time points. The transition patterns across the two countries are interesting and informative. Figure 1a demonstrates that at baseline among youth age 15, 14% had ever had sex in the Kenya cities.^2^ This increases to more than 80% among the youth who were ages 20–24 at baseline. Over the four year follow-up period, a quarter of the 15 year olds and between 37 and 58% of 16–18 year olds transition to first sex. Four years later, more than half of the 15 year olds (58%) and a third of the 16 year olds at baseline who were interviewed at both time points had remained sexually inexperienced. Figure 1b presents the transitions to first marriage in Kenya. By age 20–24, between 44 and 72% of young women were already married. A quarter of women who were ages 16–21 at baseline transition to first marriage during the four years that follow. Among women ages 15–16 at baseline, more than three-quarters remained never married by endline. Among women ages 17–18, more than half (about 57%) remain never married. Finally, Fig. 1c demonstrates the patterns for transition to first pregnancy/birth. What is most notable is that a greater percentage of women generally transition to first pregnancy/birth than transition to first marriage; conversely, a smaller percentage of women at each baseline age remain in the never pregnant/birth category than remain in the never married category. This suggests that premarital births are common in these Kenyan study cities. By age 23–24, three-quarters of women had ever had a pregnancy/birth at baseline.

Similar graphs are presented for Nigeria in Fig. 2a-c. Figure 2a demonstrates lower sexual experience at baseline at each of the ages than in Kenya.^3^ In the four-year follow-up period, between one-third and two-fifths of women ages 15–19 at baseline transition to first sex such that by the age group 20–24, more than half of the women and up to three quarters of the oldest ages had already had sex by baseline. Most notable in Fig. 2b and c is how similar the transition patterns are between first marriage and first pregnancy. In particular, in the Nigerian cities surveyed, the data indicate that these two transitions are happening at about the same age. In Nigeria, more than half (and as high as four-fifths) of 15–19 year olds have never been married or had a pregnancy/birth by the end of the four-year follow-up. Among women 20–24 at baseline, only one quarter to two-fifths of the women remain child (and marriage) free four years later.

### Multivariate results - Kenya

Table 2 presents the multivariate results of our discrete hazard models for time to first sex, marriage, and pregnancy for youth in Kenya. Two sets of analyses were performed: 1) the standard hazard models controlling for the clustered survey design for the three events; and 2) the three-equation hazard models that allow the hazards to be correlated and adjust for unobserved heterogeneity at the individual and community levels. In the methodological appendix, we provide the estimated heterogeneity parameters and an explanation of their interpretation for the models presented. The estimated heterogeneity parameters were highly jointly significant based on likelihood ratio tests and most were individually significant as well. Some factors that were significant in the unadjusted models (e.g., religion, being a student, and type of residence) were attenuated or not significant in the models that adjust for unobserved heterogeneity indicating the importance of correcting for selection in the discrete time hazards models.

**Table 2:** Multivariate findings of timing to first sex, marriage, and pregnancy corrected for heterogeneity in Kenya

	Kenya Corrected Models		
	Time to first sex	Time to marriage	Time to pregnancy
	Coef (SE)	Coef (SE)	Coef (SE)
Model age	2.51 (0.11)***	3.62 (0.22)***	4.01 (0.30)***
Model age squared	−0.05 (0.00)***	−0.07 (0.01)***	−0.07 (0.01)***
Education level			
None/Nursery	0.88 (0.30)**	2.63 (0.61)***	2.84 (0.67)***
Primary	0.64 (0.16)***	1.69 (0.36)***	2.36 (0.37)***
Post-primary	0.53 (0.22)*	1.81 (0.47)***	2.68 (0.47)***
Secondary	0.04 (0.12)	0.60 (0.30)*	1.14 (0.32)***
College/Univerity	ref	ref	ref
Religion			
Catholic (ref)	ref	ref	ref
Protestant	−0.28 (0.11)*	−0.03 (0.22)	−0.30 (0.25)
Muslim	−0.38 (0.21)+	0.46 (0.38)	−0.03 (0.45)
City			
Nairobi (ref)	ref	ref	ref
Mombasa	−0.20 (0.15)	−0.32 (0.29)	−0.47 (0.32)
Kisumu	0.83 (0.12)***	0.79 (0.21)***	1.43 (0.28)***
Machakos	−0.32 (0.17)+	−0.57 (0.33)+	−0.42 (0.35)
Kakamega	0.46 (0.13)***	0.79 (0.24)***	1.01 (0.25)***
Student	−0.31 (0.74)	−4.40 (1.88)*	0.82 (2.00)
Religiosity (strongly religious)	−0.31 (0.08)***	−0.10 (0.14)	−0.24 (0.18)
Read newspapers	−0.46 (0.12)***	−0.73 (0.26)**	−0.81 (0.29)**
Read magazines	−0.18 (0.10)+	−0.70 (0.20)***	−0.61 (0.25)*
Listen to radio	−0.17 (0.17)	−0.43 (0.35)	−0.70 (0.45)
Watches TV	−0.15 (0.09)+	−0.65 (0.15)***	−0.58 (0.16)***
Visits other cities in 12 months	0.24 (0.08)**	0.24 (0.17)	0.28 (0.19)
Visits rural areas in 12 months	0.09 (0.08)	0.49 (0.16)**	0.38 (0.18)*
Wealth group			
Poorest (ref)	ref	ref	ref
Poor	−0.15 (0.10)	−0.19 (0.21)	−0.11 (0.22)
Middle	−0.09 (0.12)	0.38 (0.24)	0.29 (0.25)
Rich	−0.76 (0.18)***	−0.80 (0.40)*	−1.19 (0.43)**
Richest	−0.88 (0.14)***	−1.67 (0.34)***	−1.72 (0.33)***
Type of residence			
Formal/non-slum (ref)	ref	ref	ref
Slum area	0.14 (0.12)	0.24 (0.23)	−0.01 (0.27)
Peri urban area	0.10 (0.13)	−0.25 (0.23)	0.25 (0.25)
Interaction: model age*student	−0.02 (0.04)	0.08 (0.10)	−0.18 (0.10)+

+*p* ≤ 0.10; *p ≤ 0.05; **p ≤ 0.01; ***p ≤ 0.001

The tables present only the adjusted models (contact first author for the unadjusted results). Briefly, Table 2 for Kenya demonstrates that older age is associated with a greater probability of transition to first sex, first marriage, and first pregnancy; however, the age-squared variable is negative and significant indicating that the age (or duration) effect diminishes over time. Compared to women with college or university-level education, women with lower levels of education or no education were significantly more likely to transition at each age. Women from Kisumu and Kakamega were significantly more likely to transition to first sex, first marriage, and first pregnancy at each age than were women from Nairobi. Further, women from the highest wealth groups compared to women in the lowest wealth group were less likely to transition at each age. In addition, women who were strongly religious and women who read newspapers were less likely to transition to first sex at each age and women who read newspapers, read magazines, and watch television were less likely to transition to first marriage or first pregnancy than their peers.

Table 3 presents the further analysis for Kenya of a two equation model for each outcome (first marriage and first pregnancy). The first equation presents the selection into first sex and the second equation examines the time to first marriage (or pregnancy) after first sex. The variable of particular interest to this analysis is the time since first sex and the association with the transition to first marriage and pregnancy conditional upon first sex having occurred. The endogeneity of this variable is controlled for by the joint estimation. This table presents the corrected models for the time to first marriage and first pregnancy equations conditional on transition to first sex. We re-estimated the time to first sex model as part of the set of equations to control for endogeneity, however, the first sex results are not shown as they were similar to those shown earlier including only exogenous right-hand-side variables. Table 3 demonstrates that for the most part, similar factors are associated with the transition to first marriage among women who transitioned to first sex as compared to all women.

**Table 3:** Multivariate findings of timing to first marriage and timing to first pregnancy after transition to first sex corrected for heterogeneity in Kenya

	Time to first marriage	Time to first pregnancy
	Corrected	Corrected
	Coef (SE)	Coef (SE)
Model age	1.29 (0.41)**	0.57 (0.11)***
Model age squared	−0.03 (0.01)**	−0.01 (0.00)***
Education level		
None/Nursery	2.24 (0.72)**	1.44 (0.33)***
Primary	1.28 (0.34)***	1.22 (0.13)***
Post-primary	1.25 (0.47)**	1.46 (0.24)***
Secondary	0.54 (0.15)***	0.73 (0.10)***
College/University (ref)	ref	ref
Religion		
Catholic (ref)	ref	ref
Protestant	0.16 (0.09)+	0.05 (0.06)
Muslim	0.80 (0.26)**	0.31 (0.13)*
City		
Nairobi (ref)	ref	ref
Mombasa	−0.05 (0.14)	−0.12 (0.09)
Kisumu	0.19 (0.13)	0.24 (0.07)**
Machakos	−0.23 (0.16)	−0.03 (0.11)
Kakamega	0.35 (0.15)*	0.24 (0.10)*
Student	−5.53 (1.66)***	−0.39 (0.81)
Religiosity (strongly religious)	0.10 (0.08)	0.02 (0.06)
Read newspapers	−0.24 (0.11)*	−0.08 (0.07)
Read magazines	−0.16 (0.11)	−0.09 (0.07)
Listen to radio	−0.01 (0.13)	−0.14 (0.08)+
Watches TV	−0.25 (0.14)+	−0.08 (0.06)
Visits other cities in 12 months	0.03 (0.10)	−0.04 (0.06)
Visits rural areas in 12 months	0.39 (0.09)***	0.13 (0.06)*
Wealth group		
Poorest (ref)	ref	ref
Poor	−0.20 (0.12)	0.01 (0.08)
Middle	0.13 (0.14)	0.10 (0.07)
Rich	−0.17 (0.15)	−0.10 (0.11)
Richest	−1.03 (0.32)**	−0.44 (0.11)***
Type of residence		
Formal/non-slum (ref)	ref	ref
Slum area	0.22 (0.14)	−0.08 (0.07)
Peri urban area	0.16 (0.15)	0.21 (0.08)*
Interaction: model age*student	0.19 (0.08)*	−0.04 (0.04)
Time since first sex (years)	−0.08 (0.06)	−0.06 (0.02)**

+*p* ≤ 0.10; *p ≤ 0.05; **p ≤ 0.01; ***p ≤ 0.001

The main distinction we find in the comparison between the uncorrected (not shown) and corrected models of time to first marriage is that the time since first sex variable is negative and significant in the uncorrected model, however, this variable is no longer significant in the model that corrects for unobserved heterogeneity among young people who have transitioned to first sex. Table 3 also shows similar models for the transition to first pregnancy among those who transitioned to first sex. These results demonstrate that in the corrected model, the longer the time since first sex, the less likely that the young person transitions to first pregnancy at each age (this was also significant in the uncorrected model – not shown).

#### Multivariate results – Nigeria

Table 4 presents the heterogeneity adjusted models for the analyses of time to first sex, first marriage, and first pregnancy for the Nigeria sample. In Nigeria, fewer demographic factors are associated with the transitions as compared to Kenya. As expected, older age (or duration of observation) is associated with all of the transitions and there is a negative effect of the squared term suggesting that this age (or duration) effect diminishes over time. The other factors that are related to the transition to each of the outcomes are education (higher education, less likely to transition) and wealth (higher wealth, less likely to transition). In the transition to first marriage and pregnancy, youth who are Muslim are significantly more likely to transition at each age. Finally, women from Zaria are more likely to transition to first marriage and first birth at each age than women from Abuja while women from Benin and Ilorin are less likely to transition to first marriage at each age.

**Table 4:** Multivariate findings of timing to first sex, marriage, and pregnancy corrected for heterogeneity in Nigeria

	Nigeria Corrected Models		
	Time to first sex	Time to marriage	Time to pregnancy
	Coef (SE)	Coef (SE)	Coef (SE)
Model age	2.84 (0.12)***	3.48 (0.20)***	4.06 (0.21)***
Model age squared	−0.06 (0.00)***	−0.06 (0.05)***	−0.08 (0.00)***
Education level			
None/Quranic	ref	ref	ref
Primary	−0.48 (0.18)**	−0.83 (0.31)**	−0.35 (0.32)
Junior secondary	−1.70 (0.20)***	−3.05 (0.39)***	−2.58 (0.37)***
Senior secondary	−2.26 (0.23)***	−4.19 (0.48)***	−3.80 (0.43)***
Higher	−2.55 (0.24)***	−5.23 (0.46)***	4.87 (0.42)***
Religion			
Catholic (ref)	ref	ref	ref
Protestant	−0.19 (0.23)	0.60 (0.47)	0.79 (0.44)+
Muslim	0.07 (0.21)	2.58 (0.33)***	2.44 (0.35)***
City			
Abuja (ref)	ref	ref	ref
Benin City	0.19 (0.25)	−1.42 (0.57)*	−1.06 (0.54)+
Ibadan	0.08 (0.25)	−0.66 (0.59)	−0.17 (0.56)
Ilorin	−0.19 (0.23)	−1.61 (0.55)**	−0.76 (0.51)
Kaduna	0.06 (0.21)	0.43 (0.45)	0.54 (0.41)
Zaria	0.22 (0.19)	1.25 (0.45)**	1.02 (0.42)*
Religiosity (strongly religious)	−0.16 (0.14)	−0.15 (0.29)	−0.14 (0.29)
Read newspapers	−0.21 (0.16)	−0.64 (0.33)+	−0.59 (0.32)+
Read magazines	0.13 (0.23)	−1.15 (0.52)*	−0.89 (0.53)+
Listen to radio	0.16 (0.13)	0.23 (0.27)	0.07 (0.29)
Watches TV	−0.21 (0.13)	−0.12 (0.24)	−0.06 (0.25)
Visits other cities in 12 months	0.19 (0.13)	0.36 (0.27)	0.43 (0.28)
Visits rural areas in 12 months	0.09 (0.13)	0.22 (0.24)	0.32 (0.24)
Wealth group			
Poorest (ref)	ref	ref	ref
Poor	−0.26 (0.18)	−0.27 (0.34)	−0.29 (0.34)
Middle	−0.44 (0.16)**	−0.73 (0.32)*	−0.74 (0.31)*
Rich	−0.82 (0.17)***	−1.49 (0.36)***	−1.53 (0.34)***
Richest	−1.51 (0.22)***	−2.72 (0.47)***	−2.93 (0.46)***

+*p* ≤ 0.10; *p ≤ 0.05; **p ≤ 0.01; ***p ≤ 0.001

Table 5 presents the corrected models for the time to first marriage and the time to first pregnancy, among young women who ever had sex for Nigeria. In the time to first marriage model among youth who have had first sex, the city-level factors have a stronger and more significant effect such that women from Benin City, Ibadan, and Ilorin are significantly less likely to get married at each age after first sex than women in Abuja. Conversely, women from Zaria are significantly more likely to make this transition than women in Abuja. Similar results are found for the transition to first pregnancy after the transition to first sex with the addition of women from Kaduna being more likely to transition to first pregnancy as well. As found for Kenya, we find that in the uncorrected model (not shown), time since first sex was significantly associated with time to marriage, however, correcting for unobserved heterogeneity, this effect is no longer significant in Nigeria. For the time to first pregnancy, the time since first sex is negative and significant in the corrected model.

**Table 5:** Multivariate findings of timing to first marriage and timing to first pregnancy after transition to first sex corrected for heterogeneity in Nigeria

	Time to first marriage	Time to first pregnancy
	Corrected	Corrected
	Coef (SE)	Coef (SE)
Model age	0.65 (0.28)*	0.44 (0.11)***
Model age squared	−0.00 (0.00)	−0.01 (0.00)**
Education level		
None/Quranic	ref	ref
Primary	−0.23 (2.01)	0.10 (0.13)
Junior secondary	−2.13 (1.53)	−0.34 (0.16)*
Senior secondary	−3.23 (1.78)+	−0.92 (0.16)***
Higher	−5.02 (1.81)**	−1.76 (0.20)***
Religion		
Catholic (ref)	ref	ref
Protestant	1.39 (0.77)+	0.54 (0.15)***
Muslim	4.39 (0.84)***	1.49 (0.15)***
City		
Abuja (ref)	ref	ref
Benin City	−1.93 (0.52)***	−0.82 (0.15)***
Ibadan	−1.68 (0.52)**	−0.45 (0.15)**
Ilorin	−2.08 (0.38)***	−0.49 (0.15)**
Kaduna	0.77 (0.61)	0.40 (0.15)**
Zaria	3.80 (1.91)*	0.69 (0.16)***
Religiosity (% strongly religious)	−0.18 (0.33)	0.03 (0.07)
Read newspapers (% yes)	−0.40 (0.34)	−0.19 (0.12)
Read magazines (% yes)	−1.37 (0.53)*	−0.51 (0.12)***
Listen to radio (% yes)	−0.02 (0.32)	−0.04 (0.07)
Watches TV (% yes)	0.22 (0.35)	0.10 (0.09)
Visits other cities in 12 months (% yes)	−0.03 (0.26)	0.06 (0.08)
Visits rural areas in 12 months (% yes)	0.34 (0.63)	0.16 (0.09)+
Wealth group		
Poorest (ref)	ref	ref
Poor	−0.41 (0.25)	0.08 (0.09)
Middle	−0.42 (0.40)	0.01 (0.10)
Rich	−0.98 (0.45)*	−0.19 (0.11)+
Richest	−1.40 (0.44)**	−0.47 (0.13)***
Time since first sex (years)	0.00 (0.20)	−0.06 (0.03)*

+*p* ≤ 0.10; *p ≤ 0.05; **p ≤ 0.01; ***p ≤ 0.001

## Discussion

This paper uses recently collected longitudinal data in select urban sites in Kenya and Nigeria to examine key sexual and reproductive health transitions among youth. Our results demonstrate that in both countries, the majority of youth have transitioned to first sex by age 20. In Kenya, by age 21, the majority (>50%) have transitioned to first pregnancy; transition to first marriage and pregnancy are later in the Nigeria context. Notably, the transition patterns are different across the two contexts with more premarital sex and non-marital childbearing in the Kenyan context than in Nigeria. Factors associated with these transitions in both countries include education and wealth; more educated and wealthier youth are less likely to transition at each age than their less educated and poorer counterparts. In Kenya, young people from Kisumu and Kakamega are making each of the transitions at earlier ages. In models among youth who have transitioned to first sex, the time since first sex was significant and negatively associated with the transition to first pregnancy at each age so that the longer it has been since first sex, the less likely that the sexually experienced young person becomes pregnant.

These findings are consistent with other studies that demonstrate that key sexual and reproductive health transitions take place in the adolescent and young adult years [25, 26]. Pregnancy transition during the adolescent and young adult years is common, especially for young people who are newly married and preparing to begin their families; as mentioned earlier, most adolescent and young adult pregnancies take place in union and are intended [5]. That said, in the urban Kenyan context where first sex, marriage, and pregnancy are less closely tied, especially in Kisumu and Kakamega, programs are needed that offer sexual and reproductive health information and services to young people to help them to avoid unintended pregnancies that can lead to poor social and health outcomes for the women and their children. Further, as the age at marriage becomes later in urban sub-Saharan African contexts, the risk of premarital sex and unintended pregnancies increases. Young people may resort to illegal and unsafe abortions if they experience an unintended pregnancy [9].

While our data are not specific enough to inform directly the discussion of the links between schooling, marriage, pregnancy, and drop-out rates, we do find that higher education is associated with delayed first sex, first marriage and first pregnancy. However, it is important to note that education may be endogenous in these models since it is also a choice variable for the individual and pregnancy could lead to school drop-out and less school completion. In addition, as shown in earlier studies, young women who are doing less well in school may be more likely to become pregnant rather than pregnancy being the cause for lower school attainment [16]. Our results of higher education being associated with delayed transitions are consistent with a recent analysis using Demographic and Health Survey data from multiple countries that demonstrated that on-time school enrollment for girls (i.e., around age 6), and support for quality education for girls (and boys) can lead to higher educational attainment and delayed marriage and pregnancy transitions [3]. Keeping girls in school and delaying first sex, first marriage and first pregnancy/birth results in better long-term health outcomes for women and families through increased potential for economic engagement and reduced household or individual poverty; this is important for ending poverty and ensuring healthy lives and promoting well-being for all at all ages (Sustainable Development Goals 1 and 3) [6]. Our findings demonstrate the need to target those young people in cities with earlier first marriage or pregnancy (e.g., Kakamega and Kisumu in Kenya and Zaria and Kaduna in Nigeria). Notably, there may be cultural norms that are supportive of earlier marriage/pregnancy in these contexts and thus programs need to be broad-based and address these cultural norms through engagement of community leaders, families, men, and young people. One approach that has been effective at keeping girls in schools is social welfare and cash transfer programs [18–20]; these may be worth testing in these sites that currently encourage early marriage and pregnancy.

This study has a number of strengths and limitations. A strength is the inclusion of a large sample of urban youth from a representative population-based survey in each of the study cities undertaken in 2010/2011; these youth were followed four years later. By including youth both in-school and out of school, transition patterns are more similar to the general population of the study cities compared to studies that focus specifically on school-going youth who are followed longitudinally [14]. Further, by controlling for the unobserved heterogeneity in our modelling approach, we are able to control for selectivity issues that are often ignored in similar types of analyses.

That said, this study is not without limitations. The biggest limitation is the loss to follow-up in Kenya, particularly among the sexually experienced, married, and ever pregnant young people. This may reflect greater mobility among these young people who may be changing place of residence with a spouse or at the time of a birth. For both countries, those who had made the transition prior to baseline data collection would have contributed full information to the study models. The challenge is those who had not transitioned by baseline and were lost to follow-up. If these young people are different, such as being more likely to delay a birth or have a premarital birth, this will bias our results. Unfortunately, with the data available, we are unable to assess the extent of bias in the results presented. A second limitation is that we were not able to code whether women were students in Nigeria; this information was not collected at baseline. Further, we were forced to assume that all young people started school at the same age and followed a similar trajectory with school-going; in reality, this is not the case as some students start and stop school while others might have to repeat a grade. Future longitudinal studies that start with a large, representative sample of young people can better measure these factors from the first time point; because our study was part of a larger evaluation that included women of all reproductive ages, additional details for the young people on their education patterns were not collected. Finally, this study used information at baseline on age of transitions (for those who already transitioned) and then followed longitudinally the transitions for those who were found four years later. This longitudinal design is limited because not all young people will transition in the follow-up period. A longer follow-up duration is required to fully examine the transition patterns of a cohort; this is what has been done in larger longitudinal studies of young people [15, 27].

Future analyses with these data will include examination of the intentionality of first pregnancies among young people who became pregnant in our observation period. This will permit a determination of whether non-marital pregnancies experienced in the follow-up period, especially in Kenya, were reported to be intended or unintended. This type of information is useful for understanding if there are unmet needs for family planning – i.e., women wanted to avoid the birth – and/or if there are other programmatic needs that include promoting delayed age at marriage and delayed first birth among newly married young people.

## Conclusions

To conclude, we show that strategies are needed to keep young people in school as this can lead to delayed sexual and reproductive health transitions including delayed first sex, first marriage and first pregnancy/birth. Prior research from sub-Saharan Africa has demonstrated that school subsidies, support for uniforms and books, social cash transfers (conditional or unconditional), school feeding, and parental monitoring can lead to improved school enrollment and better adolescent and youth reproductive health outcomes [18–20, 28]. Less is known on programs that address other young adult women’s barriers to school enrollment and completion including lack of toilets for menstrual hygiene and violence on the way to school and at school [29]. Provision of information and counseling on family planning in schools and in other settings in urban areas where young people congregate (e.g., markets, taxi stands, apprentice workshops) is a strategic approach worth pursuing to reduce unintended pregnancies and other negative reproductive health outcomes, especially in cities where early and premarital sex is common. Promoting governments, donors, and program managers to support and implement programs that focus on delaying sexual and reproductive health outcomes until the young adult (or later) years can lead to improved health for women, their children, and communities in support of attainment of the Sustainable Development Goals, particularly goals 1 and 3.

## Methodological appendix

In the estimation strategy, rather than make a specific parametric assumption about the distribution of the *λ*’s and *μ*’s, for example, multivariate normality is often assumed, we use a variation of the discrete factor approximation [24]. Specifically, we use what Mroz [30] refers to as non-linear heterogeneity where mass points are estimated for each equation along with a common set of probabilities. This form for the discrete factor model allows for very general patterns of correlation across the set of equations and has been shown to work very well in extensive sets of Monte Carlo experiments [30, 31]. Specifically, the Monte Carlo results show that the discrete factor method works almost as well as methods that assume multivariate normality for the error term distribution when the true error distribution is in fact multivariate normal and considerably better than methods that assume multivariate normality when the true distribution is not normal. Note that the model specification is highly non-linear and that even though there are exogenous regressors in the model, the model is still identified due to the non-linearity and the presence of time varying exogenous variables [32].

Table 6 provides the estimated heterogeneity parameters for both Kenya and Nigeria. Note that the estimation method sets the first mass point equal to zero for both the community and individual level heterogeneity since all the mass points and the constant are not separately identified. The number of mass points for both community and individual heterogeneity was determined empirically by adding mass points until the likelihood function improved by less than the number of additional parameters estimated [28]. The result was four community and six individual mass points for Kenya and three community and five individual level points for Nigeria. As can be seen in the table, most of the mass points are individually significant. We also performed likelihood ratio tests of the null hypothesis that the heterogeneity parameters were jointly zero. For both Kenya and Nigeria, the null hypothesis was strongly rejected with p values close to zero. Interpreting the estimated effects is basically the same as interpreting a set of dummy variables. For example, for Kenya, the second mass point has a probability weight of approximately 0.60 and large negative mass points for all three transitions, indicating that a large group of women were unlikely to make the transitions regardless of the values for the observed variables in the model. It is interesting that many of the large negative mass points for Nigeria are individual level also indicating groups of women not likely to make the transitions regardless of the values of observed covariates. Controlling for heterogeneity also causes the effects of several of the estimated coefficients of the observed variables to change as would be expected given the high level of selection on unobservables. For example, in the Nigeria unadjusted model for transition the first sex, the effect of being strongly religious on reducing the likelihood of transitioning to first sex at each age was significant; however, it is not significant with heterogeneity adjustments. This suggests that the self-selected sample over represents strongly religious youth who make a later transition and controlling for this diminishes the effect.

**Table 6:** Estimated heterogeneity parameters (standard errors) for Tables 3 and 4 controlling for individual- and community-level heterogeneity - Kenya and Nigeria

	Kenya Heterogeneity parameters				Nigeria Heterogeneity parameters		
Kenya Probability weights	Time to first sex	Time to marriage	Time to pregnancy	Nigeria Probability weights	Time to first sex	Time to marriage	Time to pregnancy
Community-Specific				Community-Specific			
0.0018	0.00	0.00	0.00	0.4053	0.00	0.00	0.00
0.6038	−3.60 (0.20)	−9.03 (0.51)	−9.28 (0.44)	0.4459	0.99 (0.10)	1.79 (0.17)	1.66 (0.15)
0.3941	−2.77 (0.21)	−7.37 (0.46)	−7.48 (0.43)	0.1487	0.81 (0.31)	−1.60 (0.71)	−1.29 (0.66)
0.0003	−0.00 (0.00)	−0.01 (0.01)	−0.02 (0.02)	na	na	na	na
Individual-Specific				Individual-Specific			
0.0774	0.00	0.00	0.00	0.0338	0.00	0.00	0.00
0.3703	−1.46 (0.17)	3.11 (0.42)	−3.51 (0.40)	0.1728	−2.25 (0.22)	−3.76 (0.28)	−3.97 (0.31)
0.1852	−2.96 (0.20)	−0.37 (0.41)	−7.36 (0.51)	0.2802	−5.23 (0.29)	−9.82 (0.43)	−10.31 (0.52)
0.0643	2.00 (0.26)	9.66 (0.66)	4.08 (0.48)	0.1740	−8.25 (0.43)	−14.88 (0.67)	−15.36 (0.77)
0.2610	−0.04 (0.17)	5.94 (0.50)	−0.12 (0.39)	0.3391	−3.75 (0.25)	−6.87 (0.35)	−7.12 (0.42)
0.0418	2.30 (0.27)	2.71 (0.55)	5.12 (0.40)	na	na	na	na

**Table 7:** Estimated heterogeneity parameters (standard errors) for Tables 3 and 5 controlling for individual- and community-level heterogeneity - Kenya and Nigeria

Kenya (sex-marriage)			Kenya (sex-pregnancy)			Nigeria (sex-marriage)			Nigeria (sex-pregnancy)		
Probability weights	Time to first sex	Time to marriage	Kenya Probability weights	Time to first sex	Time to pregnancy	Nigeria Probability weights	Time to first sex	Time to marriage	Nigeria Probability weights	Time to first sex	Time to pregnancy
Community-Specific			Community-Specific			Community-Specific			Community-Specific		
0.1235	0.00	0.00	0.0966	0.00	0.00	0.6298	0.00	0.00	0.1622	0.00	0.00
0.8765	0.74 (0.23)	0.71 (0.36)	0.9034	0.92 (0.17)	0.23 (0.27)	0.2374	0.61 (1.22)	2.38 (0.33)	0.2875	1.13 (0.24)	1.11 (0.37)
na			na			0.1327	1.18 (0.40)	0.42 (2.84)	0.5502	0.15 (0.17)	1.34 (0.29)
Individual-Specific			Individual-Specific			Individual-Specific			Individual-Specific		
0.0193	0.00	0.00	0.0015	0.00	0.00	0.1025	0.00	0.00	0.0894	0.00	0.00
0.7085	−1.85 (3.11)	−3.13 (1.08)	0.0486	−7.30 (1.11)	−6.11 (3.55)	0.3307	3.43 (1.08)	8.69 (2.37)	0.4922	4.57 (0.61)	1.68 (0.57)
0.2722	−2.30 (3.43)	−5.47 (0.74)	0.9499	−5.29 (0.67)	−3.58 (1.07)	0.1544	2.85 (0.55)	12.36 (3.26)	0.3771	2.65 (0.45)	1.56 (0.54)
na			na			0.2696	1.57 (1.37)	6.55 (1.94)	0.0248	4.43 (0.69)	−1.93 (0.78)
na			na			0.1428	3.22 (0.60)	3.82 (1.21)	0.0165	1.98 (0.76)	−7.91 (1.65)

## Abbreviations

BMGF: Bill & Melinda Gates Foundation
IRB: Institutional review board
MLE: Measurement, learning & evaluation
PSU: Primary sampling unit
SRH: Sexual and reproductive health
SSA: sub-Saharan Africa
URHI: Urban reproductive health initiative

## Footnotes

1 Note that only a small percentage (<1%) of women had inconsistent responses and these women are not shown in the figures presented.

2 In the full baseline sample, 17% of the 15 year olds, 26% of the 16 year olds, and 43% of the 17 year olds had already had first sex (contact first author for full set of values).

3 There is little difference in first sex experience between the full baseline sample and the sample interviewed at both time points (contact first author for values)
